# Growth-rate distributions of gut microbiota time series

**DOI:** 10.1038/s41598-024-82882-x

**Published:** 2025-01-22

**Authors:** E. Brigatti, S. Azaele

**Affiliations:** 1 Instituto de Física, Universidade Federal do Rio de Janeiro, Av. Athos da Silveira Ramos, 149, Cidade Universitária, Rio de Janeiro, RJ 21941-972 Brazil; 2 Dipartimento di Fisica “G. Galilei”, Università di Padova, Via Marzolo 8, 35131 Padua, Italy; 3 INFN, Istituto Nazionale di Fisica Nucleare, 35131 Padua, Italy

**Keywords:** Population dynamics, Growth-rate distribution, Neutral models, Microbiota, Microbiome, Population dynamics, Ecological modelling, Nonlinear phenomena

## Abstract

Logarithmic growth-rates are fundamental observables for describing ecological systems and the characterization of their distributions with analytical techniques can greatly improve their comprehension. Here a neutral model based on a stochastic differential equation with demographic noise, which presents a closed form for these distributions, is used to describe the population dynamics of microbiota. Results show that this model can successfully reproduce the log-growth rate distribution of the considered abundance time-series. More significantly, it predicts its temporal dependence, by reproducing its kurtosis evolution when the time lag $\tau$ is increased. Furthermore, its typical shape for large $\tau$ is assessed, verifying that the distribution variance does not diverge with $\tau$. The simulated processes generated by the calibrated stochastic equation and the analysis of each time-series, taken one by one, provided additional support for our approach. Alternatively, we tried to describe our dataset by using a logistic neutral model with an environmental stochastic term. Analytical and numerical results show that this model is not suited for describing the leptokurtic log-growth rates distribution found in our data. These results support an effective neutral model with demographic stochasticity for describing the considered microbiota.

## Introduction

Quantitative studies of gut microbiota have generated an increasing and rich body of information about different features of these ecosystems. This has been made possible by the fact that nowadays these microbial communities present a more experimental accessibility than other macroscopic ecological communities, as current sequencing technologies can monitor with high temporal resolution the dynamics of abundance of bacteria^1^. Numerous studies have described some aspects of the community ecology of these systems. They outlined macroecological patterns that characterize global statistical relationships of spatial and temporal variation and taxonomical diversity^2–4^ and even their temporal changes associated with host health, diet and lifestyle^5^. However, a robust and general description of gut microbiota at the level of population dynamics is still missing. Therefore, it is interesting and important to identify quantitative statistical stylized facts which can characterize the population dynamics of gut bacteria, with the ultimate goal of pointing out the biological processes and factors governing these dynamics.

In this work we will focus on the population dynamics displayed by the different species of bacteria present in gut microbiota. A very important observable used in the description of population dynamics is the logarithmic growth rate. Given an abundance time series *x*(*t*), the log-growth rate is defined as: $g(t,\tau )=\ln {x(t+\tau )}-\ln {x(t)}.$ The time series of this quantity have been traditionally used in population dynamics for analyzing important features of the corresponding populations. Some analysis methods proposed by Royama^6^ are capable of generating clear diagnoses of their ecological features, in particular density dependence. This phenomenological approach is based on the fit of the population growth rate and it is more efficient in testing and diagnosing, rather than in modeling^7,8^.

The idea behind the use of this quantity, instead of directly considering abundance time series, is linked to the construction of an observable close to stationarity. In fact, abundances are frequently non-stationary and subject to large variations and measurement errors. It follows that it is more robust to consider the fluctuations of the observable, measured as the difference between two consecutive logarithms of the abundance. Indeed, if the process has multiplicative dynamics, logarithms are a natural choice because they eliminate underlying exponential growth trends. Finally, it is interesting to note that this observable has been traditionally associated with a discrete approximation for the instantaneous population per capita growth rate, since $\ln (x(t + 1))-\ln (x(t))\approx \frac{1}{x}\frac{dx}{dt}$ in a first, rough approximation.

In this study we will focus on the characterization of the $P(g,\tau )$, the distribution of the $g(t,\tau )$ when the process has reached stationarity, and therefore *g* does not depend on *t*. This distribution has been already studied for the microbiota by Ji et al.^9^. Moreover, it has been actively investigated not only in other ecological systems^10^, but also in finance (the return distribution)^11^, economics^12–14^ and social science^15^. There, the considered underlying observables were not population abundances but, respectively, prices, firms or fund sizes, and crimes.

Early simple multiplicative models, discrete or continuous, hypothesized that the Gaussian distribution should emerge as a typical shape modeling this distribution. In contrast, empirical studies showed that, generally, $P(g,\tau )$ are not simply characterized by Gaussian distributions, but it is common to find shapes close to the Laplace distribution^10,12,15^ or, in finance, distributions with power-law tails^11,16^. There is a second, important, statistical feature characterizing the temporal dependence of the shape of $P(g,\tau )$. $P(g,\tau )$ is markedly dependent on $\tau$ and seems to be attracted towards a Gaussian shape for increasing values of this parameter. This last behavior is well known in finance^11,14^, where it is called aggregational gaussianity and it is justified on the basis of the central limit theorem. As $g(t,\tau )$ is time additive, for independent data points, the distribution is expected to converge to a Gaussian one for large $\tau$.

In this study we will characterize $P(g,\tau )$ and its temporal dependence by using a stochastic model which describes the underlying abundance time series^17^. This method allows to analytically obtain the $P(g,\tau )$ and its temporal dependence and to compare them with the empirical data. In addition to the description of the principal features of the log-growth rate distribution, this approach suggests a specific model of population dynamics for the species present in the considered microbiota. This model is part of a class of models which describe ecological systems within a neutral framework. Neutral theories^18,19^ posit that the dominant factors that determine the structures of an ecological community are driven by the demographic randomness present in the populations, which determine their random drift. By contrast, the selection produced by the interactions among the individuals, the species identity and the environment effects are considered far less relevant. In its more universal implementation, the neutral theory of biodiversity models the organisms of a community with identical per capita death, birth, immigration and speciation rates^18^. Species are considered demographically and ecologically equivalent and characterized by the same demographic rates. Among the different models generated by these ideas, we consider a very simple and general one, based on a stochastic differential equation (SDE) which describes the dynamics of the population. It is driven by a linear drift and the noise term includes the square root of the population, which describes demographic noise^17^. This approach generates predictions at stationarity, which have been successfully applied in a variety of different systems, such as^20^. Indeed, this SDE is a paradigmatic neutral model which can be solved analytically and can be benchmarked under different conditions. At stationarity, it predicts a distribution of species abundances which is in good agreement with empirical data collected in neotropical forests and coral reefs^19,20^. The temporal dynamics of species abundances is also well captured^17^ and can be used to predict characteristic temporal scales which are empirically inaccessible. Finally, the model is amenable to spatial generalisations^21^ which generate patterns across scales very close to those observed empirically. More recently, it has been showed that it is able to capture universal features of fluctuations in disparate systems^22^, thus improving our ability to forecast rare events.

With the aim of introducing some comparisons between the statistical patterns generated by different approaches, we consider a second model recently used for describing population dynamics in microbiota^23,24^. In this approach, populations are modeled by a traditional logistic growth term, coupled with a source of environmental stochasticity implemented by a simple multiplicative term. In Section “Methods” these two models will be described in detail.

## Data

In recent times microbiome data have become increasingly accessible and a variety of different datasets have been analyzed in several studies. Here we focus on the dataset previously considered by Zaoli et al.^24^. As we are interested in the population dynamics of the bacterial abundance, and not in the characterization of the community ecology, we look for data focusing on their statistical quality (relatively high sampling frequencies and relatively few gaps), rather than worrying about their generality. For this reason, we select time-series presenting daily sampling frequency, and which display positive read counts in at least 75% of the data points. We end up with data from four healthy human individuals: 2 individuals, M3 and F4, from the Moving Pictures MP dataset^25^ (indicated as M3F4 reads), and the time-series of the post-travel period of individual A and the pre-Salmonella interval of individual B of the study of David et al.^5^ (indicated as IndAB reads). This dataset corresponds to a total of 1305 abundance time-series of bacterial operational taxonomical units (OTU) and present a length spanning from just over 4 months to around 14. There is a huge variability in their abundances. Examples of some time-series can be seen in Fig. 2. These time-series are not all stationary. The statistical properties of stationary time-series are independent of the point in time at which they are observed. In fact, in these series, the joint probability distribution of any subsequence of data points does not change with a shift in time. As we will focus our analysis on time-independent distributions and the analytical distributions obtained from the considered theoretical models are valid only at stationarity, we restrict our dataset to the stationary time-series. These series are selected by using the Dickey-Fuller test^26^ and correspond to 75% of the original dataset.

## Methods

The neutral framework can be implemented by describing the abundance dynamics *x*(*t*) with the following SDE:

1 $$\begin{aligned} dx_t= (b-x_t/a)dt+\sqrt{2D x_t}dW_t, \end{aligned}$$

where $a,b,D>0$ and $W_t$ is a standard Wiener process. The solution of this equation is a randomly fluctuating population which is drawn back to a long-term deterministic value equal to *ab*, with a correlation time determined by *a* and fluctuations controlled by the parameter *D*. The process tends to drift towards its long-term stationary value because of its deterministic decaying term. The return time at which populations come back to the stationary abundance after a disturbance is equal to *a*. This SDE is known in the literature as the Cox–Ingersoll–Ross (CIR) equation. It was first introduced by Feller for modeling population growth^27^ and, following its use to model interest rates^28^, it became very popular in the finance literature. More recently, it has been adopted for describing Neutral dynamics in ecological systems^17^ and we consider this interpretation as the theoretical underpinning of its use in this work. This equation has an explicit solution. The propagator can be also analytically obtained and the stationary distribution is given by the Gamma distribution:

2 $$\begin{aligned} P(x)= \frac{x^{\alpha -1}e^{-x/Da}}{\Gamma (\alpha )(Da)^{\alpha }}, \end{aligned}$$

where we introduce the notation $\alpha =b/D$. This closed form allows to directly compare the empirical abundance distribution obtained from stationary time-series and the one generated by the considered SDE. Despite this process has been frequently used in finance, where returns are a fundamental measure, only recently an analytical result have been obtained for describing the log-growth rate distributions $P(g,\tau )$^17^. Following Azaele et al.^17^, this distribution can be obtained starting from the conditional transition density and the stationary probability distribution, which allows to calculate the probability for a given time lag $\tau$ of the ratio $r(\tau )=x(t_0+\tau )/x(t_0),$ where $x(t_0+\tau )$, with $\tau >0$, and $x(t_0)$ are the population abundance at time $t_0+\tau$ and $t_0$, respectively. Assuming stationarity at $t_0$, the probability distribution of $r(\tau )$ does not depend on $t_0$. The logarithmic growth rate distribution is finally obtained after the change of variable $g(\tau )=\ln {r(\tau )}$ and is given by:

3 $$\begin{aligned} P(g,\tau )=C \frac{e^{g}+1}{e^{g(\alpha +1)}} \left[ \frac{4e^{2g}}{(e^{g}+1)^2e^{\tau /a}-4e^{g}}\right] ^{\alpha +1/2} \left[ {\sinh (\tau /2a)}\right] ^{\alpha +1} \frac{e^{\frac{\alpha \tau }{2a}}}{1-e^{-\tau /a}} \end{aligned}$$

where *C* is a renormalization constant equal to $\frac{2^{\alpha -1}}{\sqrt{\pi }}\frac{\Gamma (\alpha +1/2)}{\Gamma (\alpha )}$. Note that the asymptotic behavior of the tails of the distribution, for $g \rightarrow \pm \infty$ and fixed $\tau$, follow $e^{-\alpha g}$.

For $\tau \rightarrow \infty$ Eq. 3 reduces to:

4 $$\begin{aligned} P(g)=C \frac{2^\alpha e^{\alpha g}}{(e^{g}+1)^{2\alpha }} \end{aligned}$$

which, remarkably, is not dependent on *a*. Note that for $\alpha =1$, it is a logistic distribution, a well known distribution with a shape quite similar to the normal one, but with heavier tails.

An alternative model which has been recently used for describing the abundance dynamics of microbiota^23,24,29^ is the logistic model, which is described by the following SDE:

5 $$\begin{aligned} dx_t= \frac{x_t}{a}(1-x_t/K)dt+\sqrt{\frac{\sigma ^2}{a}} x_t dW_t, \end{aligned}$$

If $\sigma ^2<2$, the stationary distribution of this SDE also follows a Gamma law^24^, with mean $\langle x \rangle = K(1-\sigma ^2/2)$. Unluckily, the propagator^30,31^ cannot be used to calculate the log-growth distribution. In this case we can grasp some information about the behavior of $P(g,\tau )$ by calculating $d \ln {\frac{x}{\langle x \rangle }}$. By defining $y=\ln {\bigg (\frac{x}{K(1-\sigma ^2/2)}\bigg )}$ and following the Itô calculus, we obtain $dy=\frac{1}{x}dx-\frac{1}{2x^2}(dx)^2$, from which it follows:

6 $$\begin{aligned} dy_t=\frac{1}{a}(1-\sigma ^2/2)(1-e^{y_t})dt+\sqrt{\frac{\sigma ^2}{a}} dW_t. \end{aligned}$$

This expression, for $y\sim 0$, can be linearly approximated by:

7 $$\begin{aligned} dy_t\sim -\frac{1}{a}(1-\sigma ^2/2)y_tdt+\sqrt{\frac{\sigma ^2}{a}} dW_t, \end{aligned}$$

which is a Ornstein-Uhlenbeck process. It presents the stationary distribution $\widetilde{P}_s(y)=N\bigg (0,\frac{\sigma ^2}{2(1-\sigma ^2/2)}\bigg )$ and also a Gaussian propagator $\widetilde{P}(y,t_0+\tau | y',t_0)$ with mean $y'e^{-\frac{\tau (1-\sigma ^2/2)}{a}}$ and variance $\frac{\sigma ^2(1-e^{-\frac{2\tau (1-\sigma ^2/2)}{a}})}{2(1-\sigma ^2/2)}$.

**Fig. 1:**
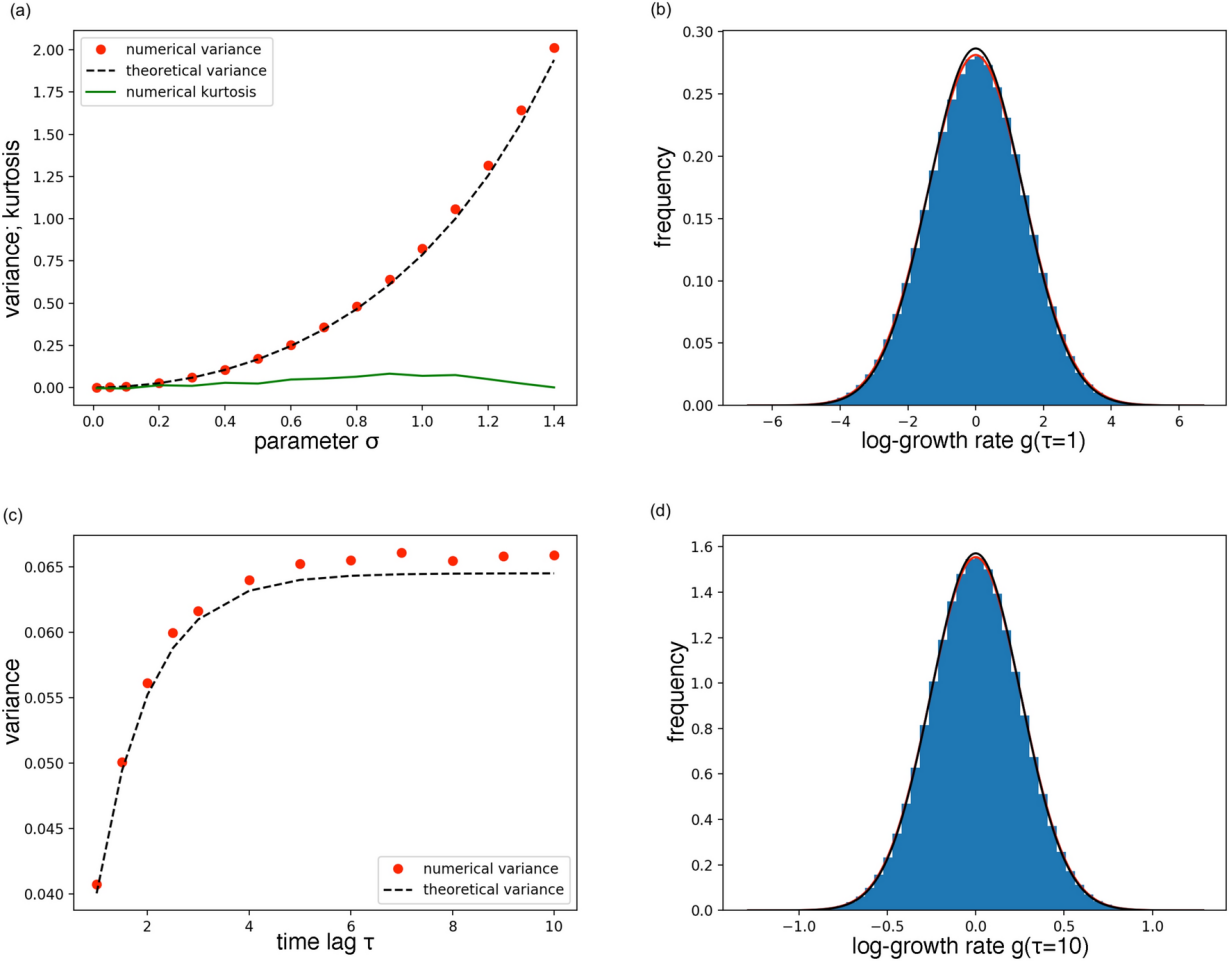
(**a**) Variance of $P(g,\tau=1 )$ as a function of $\sigma$. Red points are obtained from numerical simulations of the SDE of Eq. 5 ($K=1,a=1$). The black dashed line is the analytical approximation of Eq. 8. The solid green line represents the excess kurtosis of the distributions obtained by the numerical simulations. They are always very close to zero, showing that the distributions have always a Gaussian shape. (**b**) $P(g,\tau =1)$ for the numerical simulations with $K=1,a=1,\sigma =1.4$. The black line is the distribution obtained from the analytical approximation, the red one is a Gaussian fitting. Note that with these parameter values we are far from the regime where *P*(*y*) of Eq. 6 is Gaussian, nevertheless the approximation continues to work very well. (**c**) Variance of $P(g,\tau )$ as a function of $\tau$. Red points are obtained from numerical simulations ($K=1,\sigma =0.25$). The black dashed line is the analytical approximation. (**d**) $P(g,\tau =10)$ for the numerical simulations ($K=1,a=1,\sigma =0.25$). The black line is the analytical approximation, the red one is a Gaussian fitting.

If *x*(*t*) is the process of Eq. 5, at stationarity $P(g,\tau )=\langle \delta (g-\ln {(\frac{x(t_0+\tau )}{x(t_0)}})) \rangle =\langle \delta (g-(y-y')) \rangle$, from which $P(g,\tau )=\int _{-\infty }^{+\infty } dy \int _{-\infty }^{+\infty } dy' \delta (g-(y-y')) P(y,t_0+\tau | y',t_0) P_s(y')$, where there is no dependence on $t_0$ because we assumed stationarity. The approximation $y\sim 0$ is satisfied if $\frac{x(t)-\langle x \rangle }{\langle x \rangle }\sim 0$, which is typically true if the coefficient of variation $\sqrt{var(x)/\langle x \rangle ^2}$ is small. As the coefficient of variation of the process in Eq. 5 depends only on $\sigma$, this approximation is valid in the regime of small $\sigma$, regardless of the other parameters. Thus, in this regime, we can use the propagator and the stationary distribution from the SDE of Eq. 7 and calculate analytically the integral, obtaining:

8 $$\begin{aligned} P(g,\tau ) \approx N\left( 0,\frac{\sigma ^2\left( 1-e^{-\frac{\tau (1-\sigma ^2/2)}{a}}\right) }{(1-\sigma ^2/2)}\right) , \end{aligned}$$

where *N* specifies a Normal distribution with the corresponding mean and standard deviation. Numerical simulations confirm this conclusion and show that the approximation is fine even for all the allowed $\sigma$ values (see Fig. 1). For example, for $\sigma =1.4$, $P(g,\tau )$ is perfectly normal, as can be seen by fitting it with a generic Gaussian distribution. The difference in the variance of this generic Gaussian and the one of Eq. 8 is only $3.6\%.$ Finally, numerical simulations verify that the shape of the $P(g,\tau )$, regardless of the regimes considered, is always indistinguishable from a Gaussian one.

## Results

Here we present the study of a selection of the dataset described in Section “Data”, analysing the IndAB time-series. However, similar results are obtained when considering the whole dataset (see Supplementary Material). The first part of the investigation analyzes the ensemble of all time-series present in this selected dataset together, assuming that the same single parametrization of the CIR model is able to describe the population dynamics of all OTUs. Supposing that all OTUs do not strongly interact between each other, we can model them as a community with effective parameters, in which OTUs behave similarly. Although this assumption is only a rough approximation, the considered time series of the single OTUs can still be described by a distribution that captures the behavior of the whole community, which is in a regime of large fluctuations. This modeling approach is also supported by the results that we will present in Fig. 6. For this reason, we can hypothesize that the mixture of the single OTUs is well described by the neutral distributions for the whole community with effective parameters. Thus, for the analysis of the abundance distribution we merge the abundances of all the different OTUs present in the IndAB time-series. In the case of the log-growth rate distribution, we calculate the log-growth rates for each OTU present in the IndAB dataset and then merge the arrays produced for all OTUs into a single one, obtaining their distribution.

**Fig. 2:**
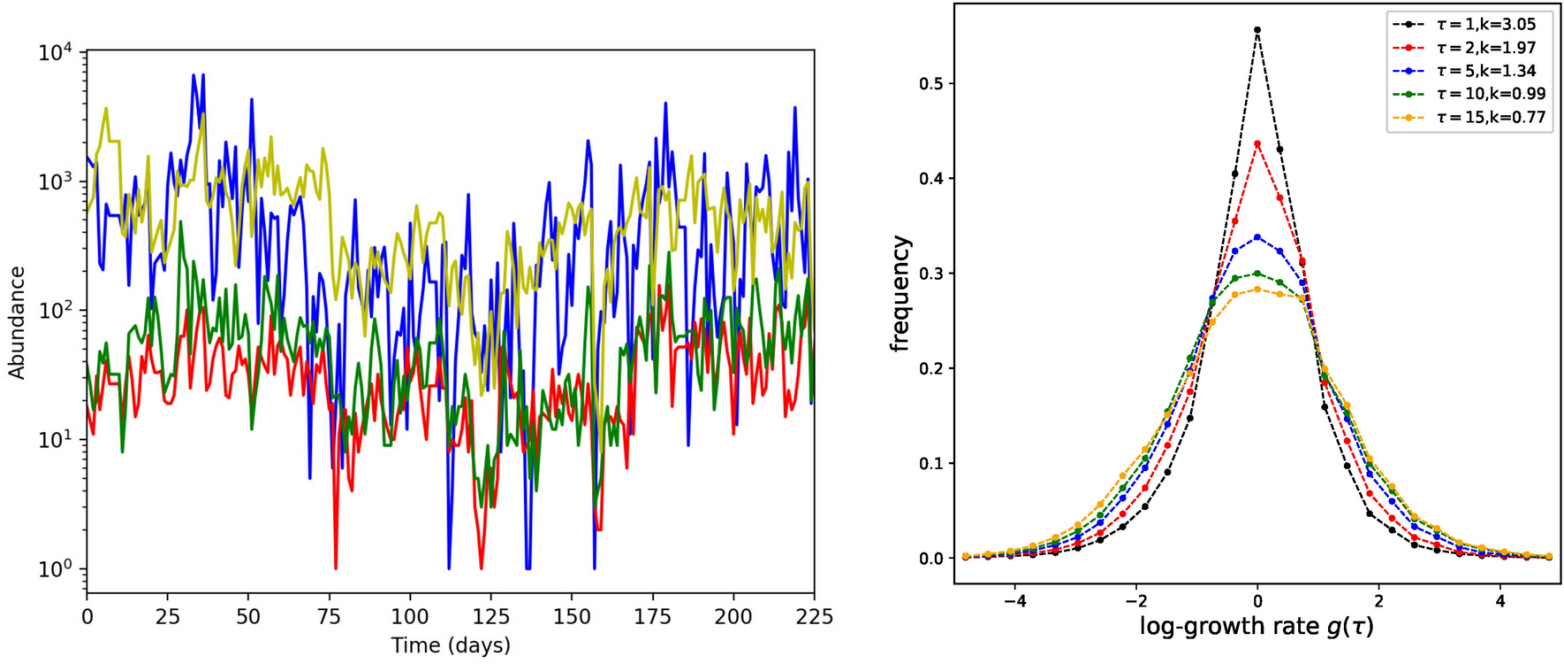
On the left: examples of some empirical abundance time-series. We can note the wide fluctuations. Actually, the coefficient of variation is practically always close to or greater than one for all the considered time series. On the right: Distribution of empirical log-growth rates measured at different time lags $\tau$. By increasing $\tau$, the distributions present lower peaks but fewer outliers. This change results in smaller excess kurtosis *k*.

First, we look at the log-growth rate distributions $P(g,\tau )$ for different values of $\tau$ (see Fig. 2). For small $\tau$ the distributions present a characteristic shape with a sharp and high peak followed by tails that decay slower than a Gaussian distribution. This shape is well characterized by the excess kurtosis ($k=\langle (\frac{x-\mu }{\sigma })^4\rangle -3$, where $\mu =\langle x \rangle$ and $\sigma ^2=\langle (x-\mu )^2\rangle$), which is obviously positive and, for $\tau =1$, close to 3. Increasing $\tau$ the excess kurtosis becomes noticeably smaller: peaks decrease and tails become more similar to those of a Gaussian distribution.

**Fig. 3:**
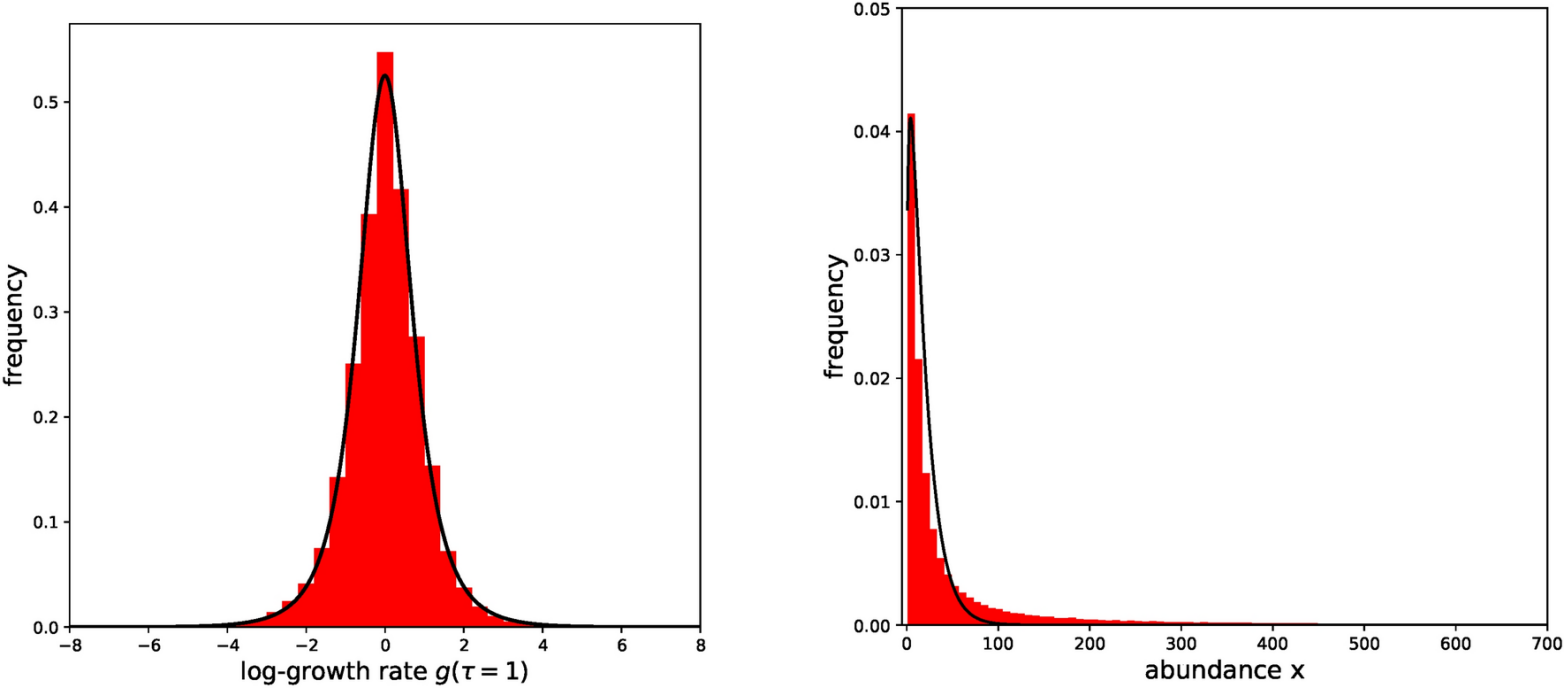
On the left: fit of $P(g,\tau =1)$ using the analytical expression of Eq. 3. On the right: the stationary abundance distribution fitted using Eq. 2 once fixed $\alpha$ with the results obtained by the estimation of the $P(g,\tau =1)$ parameters. The solid black lines represent the fits, the red histograms show the empirical data.

We focus on $P(g,\tau =1)$ and fit this distribution with Eq. 3. Parameters are estimated using the Maximum Log-likelihood method, which provides the values $\alpha =b/D=1.30\pm 0.01$ and $a=2.70\pm 0.02$. The fit is excellent, as can be visually confirmed in Fig. 3. We numerically computed the excess kurtosis of the fitted distribution, obtaining a value of 3.00, which very well approximates the empirical one (3.05). Note that the model described by Eq. 5 can not reproduce the empirical $P(g,\tau =1)$. This is because it can generate only distributions with negligible excess kurtosis (Gaussian distributions), while the empirical one is positive and large. Since in our study we are interested in identifying a departure of $P(g,\tau )$ from a Gaussian shape focusing on the behavior of the tails, the analysis of the kurtosis is a sufficient test for identifying non-normality. We have also performed statistical normality tests. The Jarque-Bera and the Anderson-Darling tests give a practically null p-value, excluding the null hypothesis of normal distribution. A fit with a normal distribution provides a poor result as well (see Supplementary material).

To find further support for our hypothesis, we fit experimental abundance data coming from all our time-series with the stationary distribution of Eq. 2 once fixed $\alpha$ with the results obtained by the estimation of the $P(g,\tau =1)$ parameters. We obtained $D\cdot a = 14.06\pm 0.08$. Gamma distributions have been already used for describing the abundance of microbiota^4,23,24^. Bearing in mind that our fit was obtained after fixing $\alpha$, and therefore by varying only the parameter $D\cdot a$, we can consider its quality acceptable.

**Fig. 4:**
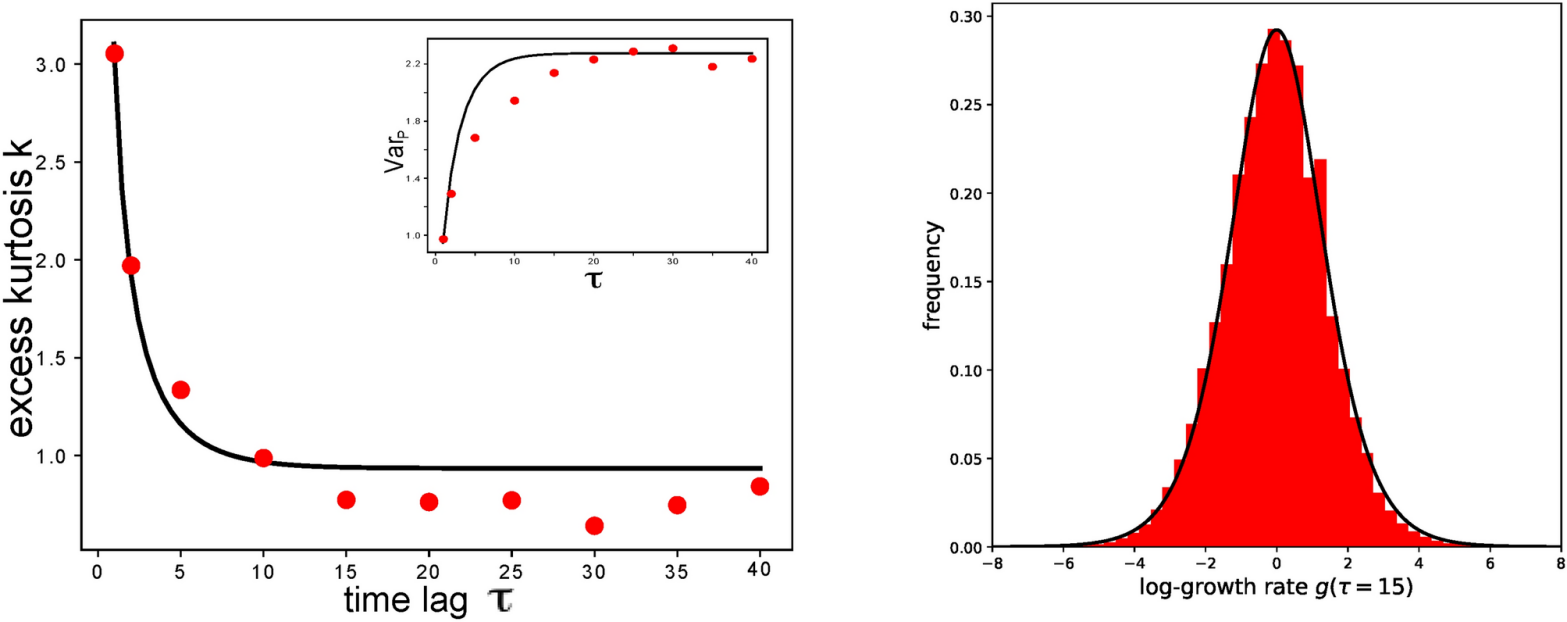
On the left: excess kurtosis as a function of $\tau.$ In the inset, lag-dependence of the variance of $P(g,\tau ).$ The solid lines are the values predicted by using Eq. 3, using the parameter values estimated at $\tau =1$. Red points are obtained from empirical data. On the right: Solid line represents the analytical distribution for $\tau\gg1$ (see Eq. 4) when using $\alpha$ estimated at $\tau =1$. This curve approximates well $P(g,\tau =15)$ obtained from the experimental dataset.

Next, we test how our model can well describe the temporal shift of the shape of the $P(g,\tau )$ towards a distribution with smaller excess kurtosis. We do that by comparing the analytical prediction produced by Eq. 3 with our experimental data. Predictions are generated calculating the excess kurtosis at different $\tau$ values from the analytical distribution with the parameters fixed by the estimation at $\tau =1$. Taking into account that kurtosis estimation is very sensitive to outliers, the excess kurtosis obtained from empirical data are very well matched by the analytical prediction, as can be seen in Fig. 4. In the same figure, the variance of $P(g,\tau )$, indicated as Var$_{P}$, is displayed as a function of $\tau$. Empirical data grow and then saturate to a limit value. Predictions are produced using the same approach used for the excess kurtosis, and capture very well the value reached at saturation.

By using the $\alpha$ value estimated at $\tau =1$ Eq. 4 can predict the $P(g,\tau )$ for $\tau \rightarrow \infty$, which is a very good approximation for every distribution with $\tau\gg1$. The result is displayed in Fig. 4.

**Fig. 5:**
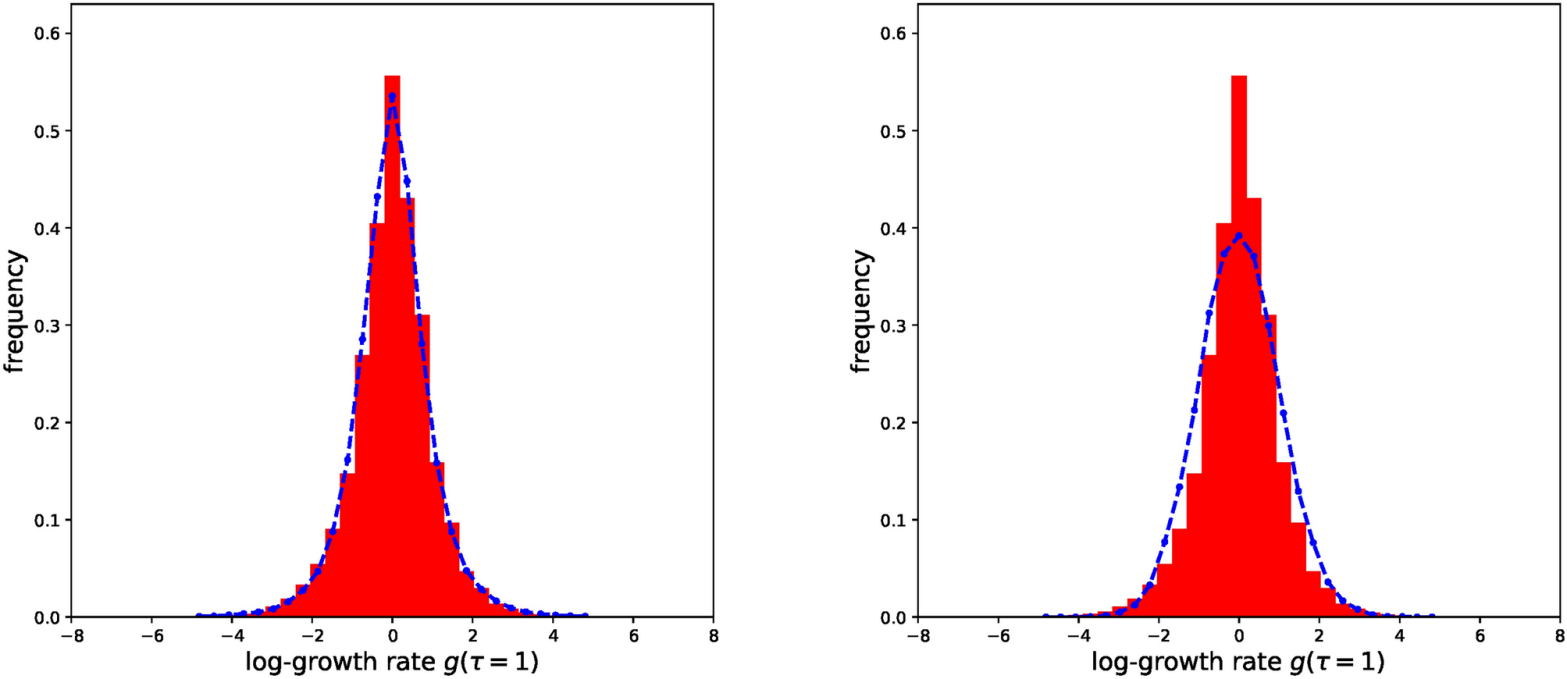
On the left: The red histogram represents the empirical $P(g,\tau =1)$, the blue dashed line shows the distribution obtained from simulations of the neutral model of Eq. 1, calibrated by using a scheme based on the log-growth distribution and the stationary abundance distribution. On the right: The red histogram represents the empirical $P(g,\tau =1)$, the blue dashed line shows the distribution obtained from simulations of the logistic model of Eq. 5. In this simulation, $\sigma$ and *K* parameters were calibrated using the stationary distribution, *a* using $P(g,\tau =1)$ fitted with Eq. 8 (see Supplementary Material). Note that choosing different values of *a* does not improve the fit with the empirical data. Fit quality for the two scenarios are compared by measuring the Kolmogorov-Smirnov statistic^32,33^ of the empirical samples with the simulated ones. Simulations using the logistic model yield less accurate results, exhibiting a K-S statistic that is 50% higher compared to that obtained when employing the SDE described in Eq. 1.

Further support to the plausibility of the considered model for describing our dataset can be found by looking at the $P(g,\tau =1)$ produced from long simulated time-series generated by this model at stationarity. The SDE of Eq. 1 is calibrated by using the *b*/*D* and *a* values obtained from the log-growth distribution and $D\cdot a$ from the stationary abundance distribution. In Fig. 5 we can see how the simulated distribution is comparable to the empirical one.

We can perform the same test using the logistic model of Eq. 5. As can be seen in Fig. 5, data generated from simulations poorly describe the experimental ones. Even if the distribution of the simulated data generally match the scale of the dispersion of the empirical one, its shape is not close to the leptokurtic empirical distribution. This result is in accordance with our prediction of the approximation of Eq. 7, which shows that the distribution is always practically indistinguishable from a Gaussian one.

**Fig. 6:**
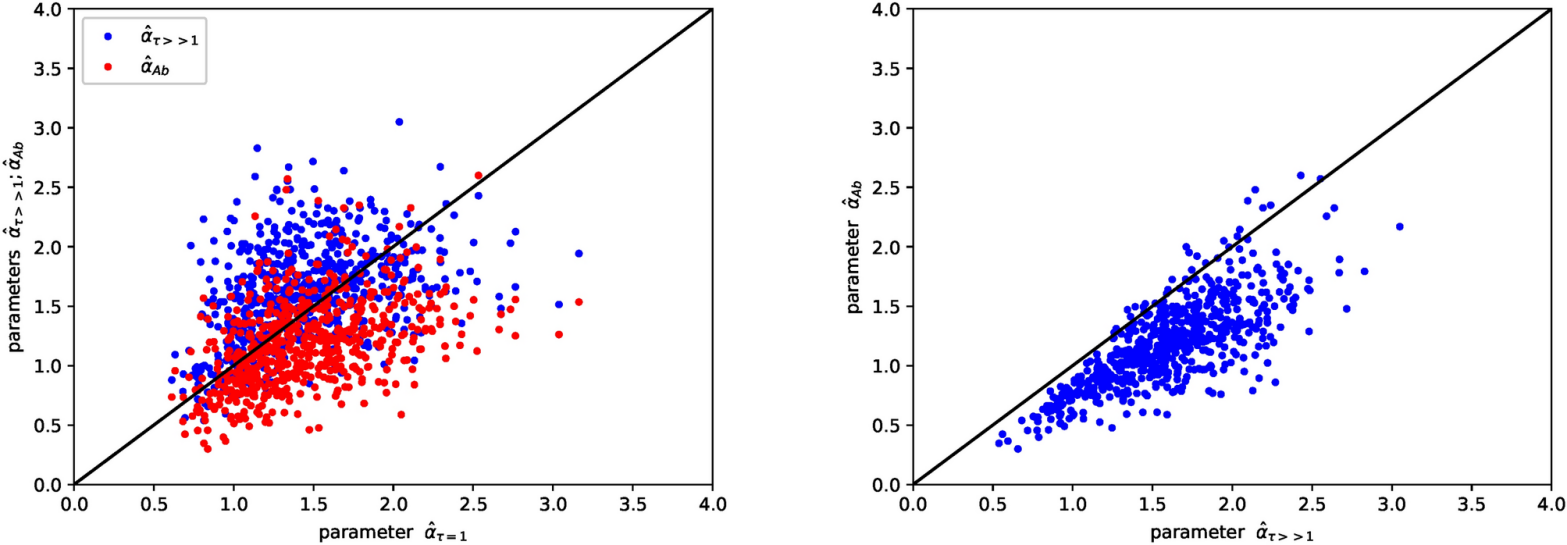
On the left: In this scatter plot blue points represent the $\hat{\alpha }_{\tau =1}$ ($\alpha$ estimated from $P(g,\tau =1)$), versus $\hat{\alpha }_{\tau\gg1}$ ($\alpha$ inferred from $P(g,\tau\gg1)$). Red points stand for $\hat{\alpha }_{\tau =1}$ versus $\hat{\alpha }_{Ab}$ ($\alpha$ estimated from the stationary abundance distribution). The Pearson correlation is equal to 0.40 and 0.43, respectively. On the right: The scatter plot of $\hat{\alpha }_{\tau\gg1}$ versus $\hat{\alpha }_{Ab}$. The Pearson correlation is equal to 0.76. The solid lines are $x=y$.

In the following we analyse each time-series taken one by one. In this case we do not assume that they are independent, even though the parameters that we fit are effective, thus possibly including the effect of interactions. This analysis allows to verify the plausibility of the neutral hypothesis and to check the consistency of the different methods used to estimate the parameter values using the model of Eq. 1. In this approach, we independently fit the log-growth rate distribution for $\tau =1$ (2 fitting parameters: $\alpha$ and *a*), the log-growth rate distribution for $\tau\gg1$ (1 fitting parameter: $\alpha$) and the stationary abundance distribution (2 fitting parameters: $\alpha$ and $D\cdot a$). For each time series, we compare the three independently estimated values of $\alpha$, which we indicate as $\hat{\alpha }_{\tau =1}$, $\hat{\alpha }_{\tau\gg1}$ and $\hat{\alpha }_{Ab}$. Results are shown in Fig. 6. Despite the dispersion of the points, which accounts for the heterogeneity of the time series, the estimated values are distributed close to the line $x=y$. Taking into account the size and noise of the time-series, the result suggests that the different approaches used to estimate the parameter values are consistent. A small systematic deviation between $\hat{\alpha }_{\tau\gg1}$ and $\hat{\alpha }_{Ab}$ can suggests that the estimated $\hat{\alpha }_{\tau\gg1}$ are in general larger than the $\hat{\alpha }_{Ab}$.

This analysis supports the validity of applying the approximation that all OTUs behave according to Eq. 1 with effective parameters. In fact, the mean value of $\hat{\alpha }_{\tau =1}$ is equal to $1.45\pm 0.41$ and the estimation of *a* coming from the analysis of each time-series taken one by one gives $3.63\pm 2.91$ days. These values are consistent with the ones found by analyzing the ensemble of all time-series together. Moreover, the coefficients of variation of the distributions of the three $\alpha$ are always smaller or equal to 0.3, suggesting that the three distributions present a low-variance. The results presented in Fig. 6 excluded the time series which generate a $P(g,\tau =1)$ with an excess kurtosis smaller than 0.5, corresponding to the 12% of the original dataset. For these data the estimation of $\hat{\alpha }_{\tau =1}$ is inaccurate. More details can be found in the Supplementary material.

The analysis on a per-OTU basis was also carried out by using the model defined in Eq. 5. Considering all the OTUs, we run the statistical normality test of Shapiro-Wilk for each $P(g,\tau =1)$. This test produces a p-value smaller than 0.05 in 86% of the cases, showing that Eq. 5 can not reproduce these empirical distributions. The same test applied to OTUs with an excess kurtosis larger than 0.5 provides a 94% of fails.

Finally, a model selection approach was used to compare the two models. We considered the distribution of Eq. 3, generated by the CIR equation, and the Normal one, generated by the logistic SDE. To quantify the evidence supporting each model we used the Akaike information criterion (AIC), which compares models likelihoods. The AIC is calculated as follows: $AIC = 2 K- 2 L$, where *K* is the number of parameters of the model and *L* is the maximum log-likelihood. The model with the lowest Akaike information is the best supported model^34^. The analysis shows that the distribution of Eq. 3 is the best supported model for the 97% of the considered OTUs. Restricting the dataset to OTUs with distributions presenting an excess kurtosis larger than 0.5, Eq. 3 is always the best supported model. Akaike weights are displayed in the Supplementary Material.

## Discussion

Our results show that the considered neutral model with demographic stochasticity can successfully describe the log-growth rate distributions and the stationary abundance distribution derived from the stationary OTUs abundance time-series. More significantly, the model can predict the temporal dependence of the log-growth rate distribution, by reproducing the kurtosis evolution of $P(g,\tau )$ as a function of $\tau$. Furthermore, the typical shape of $P(g,\tau\gg1)$ can be independently assessed when using this approach. We can observe that this last distribution generally has a shape that is relatively close to a Gaussian one. It can be hypothesized that temporal dependencies of *g* at different times *t* produce a slight deviation from the convergence to a Gaussian shape, as suggested by aggregational Gaussianity in the case of time additive independent variables. In contrast, this heuristic reasoning based on the central limit theorem, is not useful for describing the temporal evolution of $\text {Var}_P$. If such considerations were valid, at large $\tau$, $\text {Var}_P\propto \tau$, and it would not saturate to a limit value, as it is displayed by our analysis. Note that the law $\text {Var}_P\propto \tau$ applies to diffusive models used in population dynamics^35^, but if a regulation process is present, for example in the form of a density dependence, $\text {Var}_P \propto \tau ^{2H}$ with *H*, the Hurst exponent, smaller than 0.5. This behavior has already been reported since the classical work of Keitt et al.^10^ and it can be understood by considering that regulation processes introduce in *g* anticorrelations at different times. In our case, instead, $\text {Var}_P$ grows at small $\tau$, and then saturates to a limit value. This is the first time that the idea presented in Kalyuzhny et al.^36^, which conjectures that, for stabilizing forces which drive the populations towards an equilibrium, $\text {Var}_P$ should reach a saturation point, are quantitatively confirmed by empirical data and analytical considerations.

In addition to these results, obtained comparing the analytical predictions of the model with the experimental data, we can produce some numerical results which support these outcomes. In fact, the SDE, calibrated with the parameters obtained from the fitting of the distributions, is able to generate simulated processes with a log-growth rate synthetic distribution comparable with the empirical one.

Finally, the analysis of each time-series, taken one by one, confirms the consistency of the parameters inferred by independently fitting the log-growth rate distribution, the same distribution for large $\tau$ and the stationary abundance distribution. The coefficients of variation of the distributions of these parameters are relatively small and the comparison of their mean values with the estimations derived from the analysis of the ensemble of all time-series together are also consistent. These facts support an effective neutral modeling approach in a first approximation.

The most relevant result of our work is the description of the subtle temporal dependence of the log-growth rate distribution. The importance of this result is due to the fact that the distributions that reproduce the abundance and growth rate are flexible enough for describing very different datasets. On varying its parameters, distinct distributions can be seen as a special case of the Gamma one, which can describe data which present shapes close to power-laws, exponential, and even log-normals. The expression of Eq. 3 turned out to be really versatile and has been able to reproduce log-growth rate generated by very different systems, as can be seen in^22^. The fit of these distributions is important, but not necessarily conclusive for claiming that the considered SDE, with a mean reverting linear drift and demographic noise, can account for the description of so different datasets^22^. Other features or more specific characteristics of the considered distribution should be assessed. This is achieved in our study by analyzing the temporal dependence of the $P(g,\tau )$.

Another important point raised by our analysis is the fact that the logistic model with an environmental stochastic term is not suited for describing the $P(g,\tau )$ found in the considered microbiota dataset at stationarity. Our analytical results demonstrated that for the regimes with small $\sigma$ this process produces normal $P(g,\tau )$ with a variance dependent on $\sigma$, *a* and $\tau$. Numerical simulations confirmed that this analytical approximation is good in all the regimes. For these reasons, this model can not reproduce empirical log-growth rates with leptokurtic distributions, as for the analysed data, which present a large and positive kurtosis. This is confirmed by statistical normality tests, which rejected the normality hypothesis. The same test was performed for each individual OTUs, excluding the normality hypothesis in the vast majority of cases. Finally, a model selection approach showed that the Normal distribution is the best supported model, in relation to the model of Eq. 3, for only 3% of the OTUs. Note that these results only show that, at least in the current formulation, there is no support for using the $P(g,\tau )$ produced by the logistic model with a linear stochastic term. These results should not be necessarily interpreted as evidence for Eq. 1 to be more appropriate than Eq. 5 for describing the microbiome population dynamics. This is not the aim of this work and, in order to attempt to answer this question, further analyses, richer and more robust empirical data are needed.

Finally, our study suggests that neutral models can effectively describe the population dynamics of bacteria in the considered microbiota. The existent literature on this theme reported conflicting evidence. It suggests that human microbial communities are not generally neutral but a small minority of cases already demonstrated the existence of neutral processes^2,23^. These assessments were generally obtained carrying out an analysis of the characteristic of the community ecology based on macroecological statistical properties. Here, we arrived at this conclusions using a dynamical population approach. In this sense, our results, even if obtained over a limited dataset, are particularly interesting for their implications at the level of biological factors that control the dynamics of the considered populations. The models we have analysed in this paper seem to suggest that demographic noise is relatively more important than the environmental one for explaining $P(g,\tau )$. Stochastic logistic models with quenched noise (e.g., random parameters) or which encompass time-correlated stochastic terms can provide a wider variability in the temporal evolution of the population size and therefore an improved explanatory power which might better describe log-growth distributions. However, these approaches are outside the scope of the present work.

## Supplementary Information

Supplementary Information.

## Data Availability

This paper does not use original data. The datasets analysed are available from the original references which are listed in the manuscript. E.B. can be contacted to request the processed data from this study or they can be downloaded at: https://figshare.com/s/cf765721ff4f6ff7d92a.
