# Supplementary material for: Growth-rate distributions of gut microbiota time series
Source: Sci Rep. 2025 Jan 22;15:2789. doi: 10.1038/s41598-024-82882-x (PMC11754794; doi:10.1038/s41598-024-82882-x)
Supplement: Supplementary file 1 — Supplementary Information. [file 41598_2024_82882_MOESM1_ESM.pdf]

Supplementary Material for: “Growth-rate distributions of gut microbiota time series: neutral models and temporal dependence”

E. Brigatti and S. Azaele

1. Results considering all the database

The following figures and captions describe the results obtained analyzing the entire database.

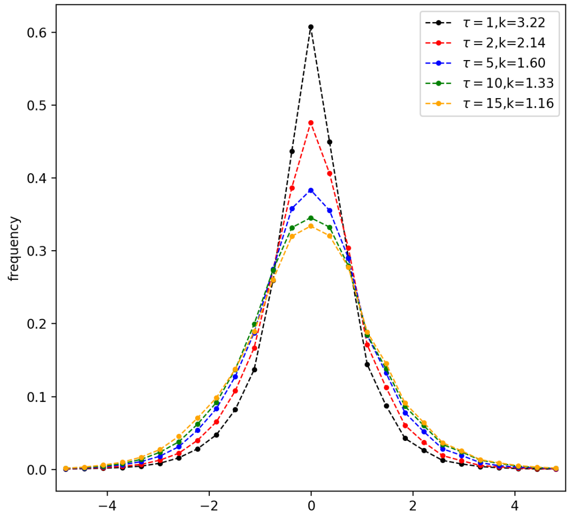

Figure 1. Distribution of log-growth rates measured at different time lags τ. By increasing τ, the distributions present lower peaks and less important tails, which corresponds to smaller excess kurtosis k.

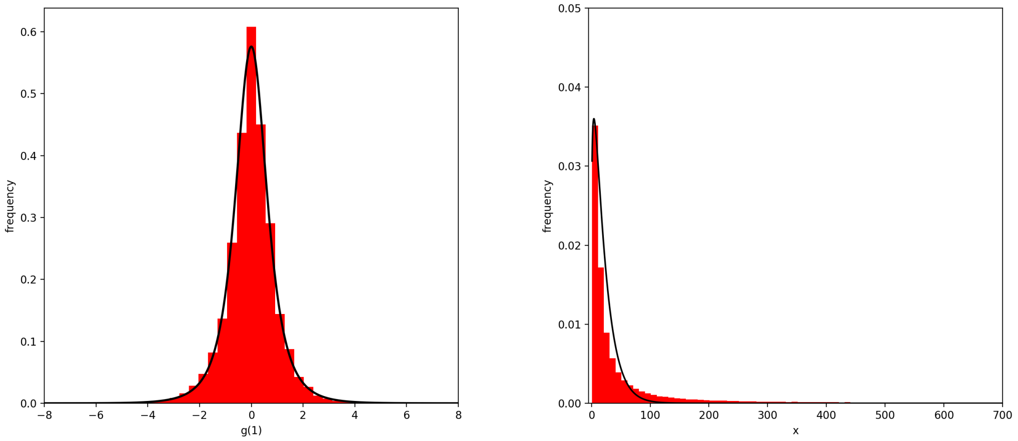

Figure 2. On the left: Fitting of $P(g, \tau = 1)$ using the analytical expression of eq. 5. Parameters are estimated using Maximum Log-likelihood and gives the values $b/D = 1.24 \pm 0.01$ and $a = 3.56 \pm 0.02$. On the right: fitting of the stationary abundance distribution using eq. 2. b/D was fixed by the previous estimation, $D \cdot a = 17.01 \pm 0.08$.

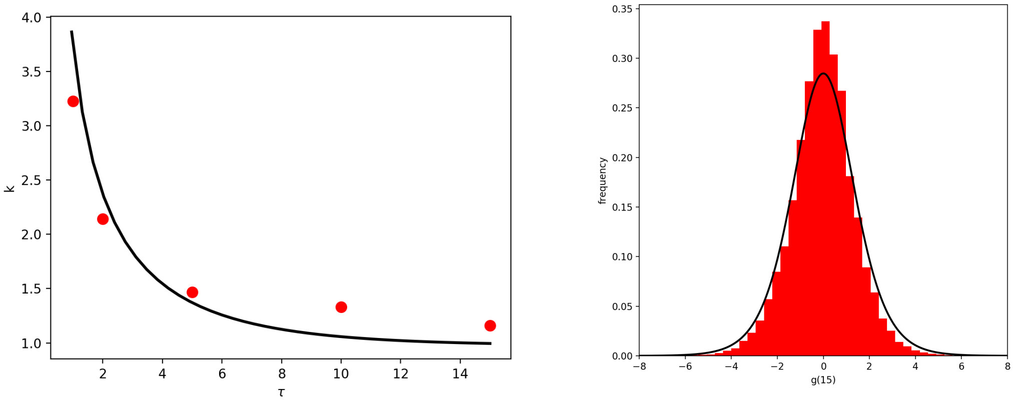

Figure 3. On the left: change of the kurtosis in dependence of τ values. The solid line are the values predicted by eq. 3, using the parameters values estimated at $\tau = 1$. Red points are obtained from empirical data. On the right: The analytical distribution for $\tau \to \infty$ (see eq. 4), using b/D estimated at $\tau = 1$, approximates $P(g, \tau = 15)$.

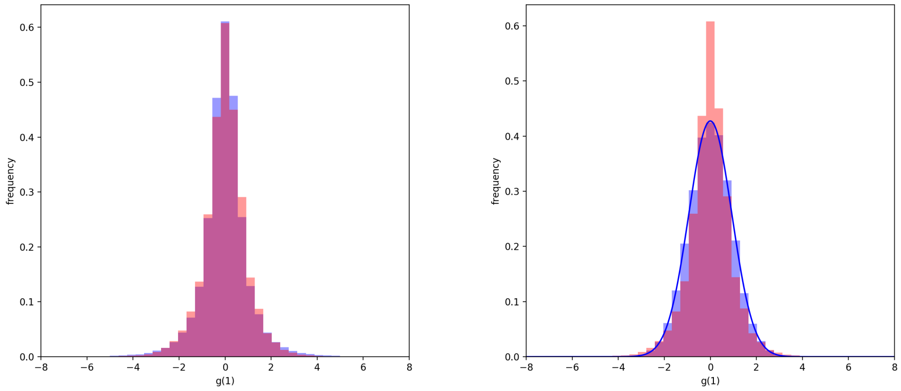

Figure 4. On the left: In red, empirical $P(g, \tau = 1)$, in blue the distribution obtained from simulations of the SDE in eq.1 calibrated using our scheme based on the log-growth distribution and the stationary abundance distribution. On the right: In red, empirical $P(g, \tau = 1)$, in blue the distribution obtained from simulations of a logistic SDE. The solid line is a Gaussian fit of the simulated data.

2. Logistic Model

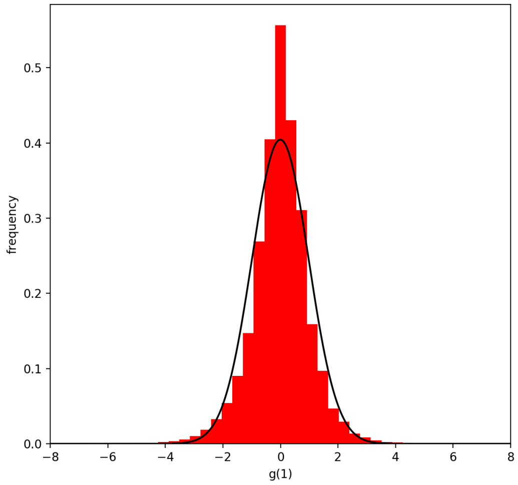

Figure 5. The histogram represents the empirical $P(g, \tau = 1)$, the solid line is a Gaussian fit obtained using results of equation 8. The fitting captures the variance of the distribution but it does not describe the leptokurtic nature of the empirical dataset.

3. Single OTUs analyses

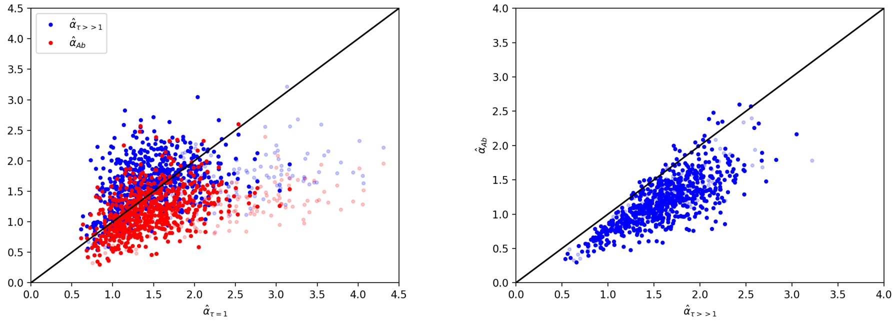

Figure 6. On the left: In this scattering plot blue points represent the $\hat{\alpha}_{\tau=1}$ (α estimated from $P(g, \tau = 1)$), versus $\hat{\alpha}_{\tau>>1}$ (α inferred from $P(g, \tau >> 1)$). Red points stand for $\hat{\alpha}_{\tau=1}$ versus $\hat{\alpha}_{Ab}$ (α estimated from the stationary abundance distribution). On the right: The scattering plot of $\hat{\alpha}_{\tau>>1}$ versus $\hat{\alpha}_{Ab}$. More transparent points represent parameters obtained from time-series which produce a $P(g, \tau = 1)$ with an excess kurtosis smaller than 0.5. The solid lines are $x = y$.

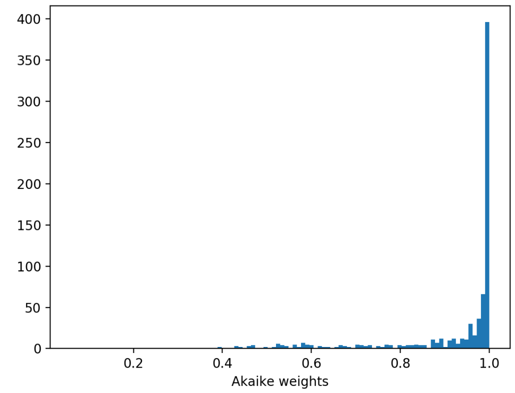

Figure 7. Akaike weights of eq. 3 (CIR model) for the log-growth rate distributions of the considered OTUs.
